# Impact of Elexacaftor‐Tezacaftor‐Ivacaftor on Quality of Life in Children With Cystic Fibrosis

**DOI:** 10.1002/ppul.71461

**Published:** 2026-01-07

**Authors:** Sara Kümmerli, Clara Fernandez Elviro, Nicolas Regamey, Anne Mornand, Mohamed Faouzi, Isabelle Rochat, Sylvain Blanchon

**Affiliations:** ^1^ Pediatric Pulmonology and Cystic Fibrosis Unit, Division of Pediatrics, Department Woman‐Mother‐Child Lausanne University Hospital and University of Lausanne Lausanne Switzerland; ^2^ Pediatric Pulmonology and Cystic Fibrosis Unit Children's Hospital of Central Switzerland Lucerne Switzerland; ^3^ Pediatric Pulmonology and Cystic Fibrosis Unit University Hospital of Geneva Geneva Switzerland; ^4^ Division of Biostatistics, Center for Primary Care and Public Health (Unisanté) University of Lausanne Lausanne Switzerland

**Keywords:** CFTR modulator, children, cystic fibrosis (CF), cystic fibrosis quality of life questionnaire (CFQ‐R), Elexacaftor‐Tezacaftor‐Ivacaftor, quality of life

## Abstract

**Objectives:**

CFTR modulators have revolutionized cystic fibrosis (CF) management by targeting the defective protein rather than its consequences. Their impact on quality of life (QoL) have been studied in numerous trials, but few data are available on QoL in patients receiving Elexacaftor‐Tezacaftor‐Ivacaftor (ETI), notably in children given its recent authorization for this age group. We aimed to assess the impact of ETI on children's QoL.

**Methods:**

This prospective observational study included children with CF (6−17 years), assessing QoL using the CF Questionnaire Revised (CFQ‐R) before (baseline) and 3 months after (M3) starting ETI treatment for children and their caregivers.

**Results:**

We included 23 children (median [range]) age 10.2 [6−17.2] years, 13 (57%) with homozygous F508del genotype. The total QoL score at baseline ([mean (SD)] children: 74.07 [10.86]; caregivers: 73.21 [10.38]) reflected severe disease impact, particularly regarding treatment burden in the children's perspective (63.28 [21.04]) and digestive domains in the caregivers' perspective (digestive symptoms: 66.67 [17.37]; eating disorder: 67.54 [32.14]; weight: 61.40 [33.82]). At M3, there was a significant increase in reported QoL (*p* = 0.0001), particularly regarding physical domains. Emotional/social/school domains barely showed improvement. Although QoL mean scores were comparable between children and caregivers' groups, they were poorly correlated within the same family. Homozygous F508del genotype was associated with better QoL improvement at M3 compared to composite heterozygous genotypes (*p* < 0.001).

**Conclusion:**

ETI treatment has a significant impact on children's QoL, particularly in physical health domains. Other QoL domains that are not improved by ETI need to be addressed, in particular, psycho‐social components. Both children's and caregivers' perspectives must be considered for a holistic picture of children's QoL.

## 1 Introduction

Cystic fibrosis (CF) is a rare autosomal recessive genetic disease due to bi‐allelic pathogenic variants in the gene *Cystic Fibrosis Transmembrane Conductance Regulator (CFTR),* including F508del, the most common pathogenic variant. CF is characterized by persistent airway infections, malabsorption, and a large panel of systemic manifestations. Due to chronic symptoms, time‐consuming daily treatments, repeated hospitalizations, and reduced life expectancy, the impact of the disease on the quality of life (QoL) of patients and their families is significant. CFTR modulators emerged in 2010 as a new CF treatment with a direct effect on the dysfunctional protein. To date, 4 molecules are available, used alone or combined: Ivacaftor (IVA), holding the chloride channel open (called “potentiator”); Lumacaftor (LUM), Tezacaftor (TEZ), and Elexacaftor (ELX), processing, trafficking, and stabilizing the CFTR protein at the apical cell membrane (called “correctors”). Four drugs are marketed: IVA, LUM/IVA, TEZ/IVA, and ELX/TEZ/IVA (ETI) [1]. The latest generation, ETI, proven particularly efficient, is authorized in Switzerland for patients 12 years and older since February 2021, and 6 years and older since May 2022.

The impact of CFTR modulators on QoL has been studied in numerous clinical trials using the CFQ‐R (CF Questionnaire Revised), but few real‐world data on QoL in children treated with ETI are available, given its recent authorization. This is of importance considering age‐specific issues in this population, such as schooling, psycho‐affective development, and family life.

We hypothesized that ETI has a positive effect on children's QoL, assessed by patients and their caregivers. Our objectives thus were: (1) to assess the QoL change in children after 3 months of treatment with ETI and identify in which domains of life it was the most significant, (2) to compare the QoL perception as assessed by the patients and their caregivers and (3) to identify patients’ characteristics associated with QoL change in children treated with ETI.

## 2 Methods

### 2.1 Study Design and Subjects

This cohort study is nested in a multi‐center prospective study focused on children and adults with CF, starting CFTR modulators in 3 CF clinics in Switzerland (Geneva, Lausanne, Lucerne) (Ethic committee of the Vaud Canton (Regional committee of Swissethics); Agreement CER‐VD 2021‐01047). All patients and/or their legal representatives gave their informed consent. Our nested study selected children included between October 2021 and January 2023, aged 6−17 years when starting treatment with ETI (switch from another CFTR modulator or introduction of a first CFTR modulator), and having completed the QoL questionnaire at baseline.

### 2.2 Data Collection

Patient parameters were collected at two assessments: within 2 months prior to the start of ETI (baseline), and 3 months after (M3). The parameters collected at baseline included patient demographics (age, sex, ethnic group, *CFTR* variants), Body Mass Index (BMI) z‐score, medical history (pancreatic function, *Pseudomonas aeruginosa* airways colonization, number of pulmonary exacerbations in the previous year, previous CFTR modulator treatment), patient‐reported symptoms (expectoration, abdominal pain), pulmonary function (Forced Expiratory Volume in 1 s [FEV_1_] and Lung Clearance Index [LCI 2.5%]), lung imaging (bronchiectasis documented by computed tomography [CT]), sweat test and airway microbiology.

As the primary outcome, QoL was evaluated at baseline and M3 by the questionnaire CFQ‐R, and scores were calculated using the software developed by the University Hospital of Copenhagen [2, 3]. Specific questionnaires are available for patients 6−13‐year‐old (CFQ‐R_6‐13) and 14−17‐year‐old (CFQ‐R_14+). An additional questionnaire is dedicated to caregivers of patients 6−13‐year‐old (CFQ‐R_Caregiver). Each questionnaire allows to calculate a total score and specific sub‐scores related to specific QoL domains. The total score and each sub‐score are standardized on a 0−100 scale, with higher scores indicating higher QoL. The specific QoL domains explored by the questionnaires are *physical functioning, respiratory symptoms, digestive symptoms, eating disorder, weight, body image, vitality, health perception, emotional functioning, social functioning, school functioning* (global energy), *role function* (capacity to maintain his/her role/position in society), and *treatment burden* [4]. CFQ‐R_6‐13 includes sub‐scores *physical functioning*, *respiratory symptoms, digestive symptoms, eating disorder, body image, emotional functioning*, *social functioning, and treatment burden*. CFQ‐R_14+ includes 4 additional sub‐scores: *weight*, *vitality, health perception,* and *role function*. CFQ‐R_Caregiver includes the same sub‐scores as CFQ‐R_6‐13, except *social functioning,* but additionally *weight*, *vitality*, *health perception,* and *school functioning*.

### 2.3 Data Analysis

Patient characteristics were summarized at baseline. CFQ‐R total score and sub‐scores were summarized at baseline and M3, then compared using the Wilcoxon matched‐pairs signed‐rank test. Robust regression analysis was used to identify patient characteristics at baseline associated with the QoL change score between baseline and M3. The strength of the association was measured using the Beta coefficient and its *p* value. The associations with *p* < 5% were considered significant. Spearman's rank correlation coefficients were used to evaluate the correlation between the QoL perception as assessed by the patients and their caregivers. Statistical analysis was performed using STATA 18 software (StataCorp. 2023. Stata Statistical Software: Release 18. College Station, TX: StataCorp LLC.).

## 3 Results

### 3.1 Patients

We included 23 patients, whose characteristics are detailed in Table 1. Only one patient didn't evidence pancreatic insufficiency at baseline. Despite more than half of patients having bronchiectasis, only one had chronic *Pseudomonas aeruginosa* colonization. At baseline, 19 (83%) and 4 (17%) patients filled out the CFQ‐R_6‐13 and CFQ‐R_14+, respectively. At M3, 20/23 patients completed the questionnaires, including 17/19 CFQ‐R_6‐13 and 3/4 CFQ‐R_14+. All caregivers of patients aged 6−13 completed the CFQ‐R‐Caregiver at baseline (*n* = 19) and M3 (*n* = 17). Given the low number of patients 14 years and older, items restricted to that age group could not be analyzed separately.

**Table 1:** Patient characteristics at baseline (*n* = 23).

Male sex, *n* (%)	12 (52)
Age (years), median [range]	10.2 [6−17.2]
Ethnic group, *n* (%)	
Caucasian	22 (96)
African‐American	1 (4)
*CFTR* genotype, *n* (%)	
Homozygote F508del	13 (57)
Heterozygote F508del	9 (39)
Other	1 (4)
Prior CFTR modulator, *n* (%)	
IVA/LUMA	6 (26)
IVA/TEZ	1 (4)
None	16 (70)
Medical history, *n* (%)	
Pancreatic insufficiency	22 (96)
Bronchiectasis	12 (52)
Chronic pseudomonas aeruginosa colonization	1 (4)
Clinical and functional parameters, median [min−max]	
Number of pulmonary exacerbations in the previous year	1.00 [0−4]
BMI (*z*‐score)	−0.21 [−2.08 to 1.43]
FEV1 (*z*‐score)	−0.47 [−3.26 to 1.59]
LCI 2.5%	8.36 [6.32 to 12.63]
Sweat chloride	102.00 [70−128]

### 3.2 Effect of ETI on QoL

QoL evaluations with total and sub‐scores at baseline and M3 are detailed in Table 2. At baseline, children and their caregivers reported a clear impact of the disease on QoL, as demonstrated by the low total score ([mean (SD)] children: 74.07 [10.86]; caregivers: 73.21 [10.38]). The highest impact of the disease was on *treatment burden* sub‐score in children's questionnaires (63.28 [21.04]), and on *digestive symptoms, eating disorder,* and *weight* sub‐scores in caregivers' questionnaires (respectively: 66.67 [17.37]; 67.54 [32.14]; 61.40 [33.82]).

**Table 2:** Summary of CFQ‐R scores at baseline and after 3 months of ETI treatment (M3) in patients and caregivers, and test for change (baseline vs. M3) using the Wilcoxon matched‐pairs signed‐rank test.

	All children			Caregivers		
	Baseline mean (SD)	M3 mean (SD)	*p* value	Baseline mean (SD)	M3 mean (SD)	*p* value
*N*	**23**	**20**		**19**	**17**	
Total score	74.07 (10.86)	82.78 (7.23)	**0.0001**	73.21 (10.38)	82.52 (7.03)	**0.0008**
Physical functioning	79.59 (16.06)	89.03 (9.63)	**0.006**	88.60 (12.02)	94.85 (7.29)	**0.007**
Respiratory symptoms	79.23 (15.67)	90.14 (10.01)	**0.01**	84.76 (15.13)	96.73 (5.22)	**0.0003**
Digestive symptoms	68.60 (26.94)	83.33 (20.23)	**0.03**	66.67 (17.37)	77.78 (13.61)	**0.01**
Eating disorder	77.30 (27.32)	88.89 (19.41)	**0.01**	67.54 (32.14)	84.31 (19.07)	**0.001**
Body image	81.51 (18.49)	89.45 (16.31)	**0.03**	73.10 (30.16)	87.58 (29.37)	**0.026**
Emotional functioning	73.01 (14.69)	74.21 (18.01)	0.39	79.30 (12.55)	84.31(13.53)	0.09
Social functioning	72.46 (10.64)	74.40 (13.70)	0.39	NA	NA	NA
School functioning	NA	NA	NA	75.00 (16.67)	69.61 (14.71)	0.49
Treatment burden	63.28 (21.04)	76.11 (21.71)	**0.007**	70.18 (11.74)	71.90 (18.05)	0.69
Weight	NA	NA	NA	61.40 (33.82)	90.20 (22.87)	**0.001**
Vitality	NA	NA	NA	67.37 (11.31)	69.41 (8.18)	0.21
Health perception	NA	NA	NA	71.35 (12.46)	81.05 (15.09)	**0.035**

	Only children 14−17 years old		
	Baseline mean (SD)	M3 mean (SD)	*p* value
*N*	**4**	**3**	
Weight	66.67 (47.14)	66.67 (57.74)	0.5
Vitality	54.16 (33.68)	72.22 (24.06)	1
Health perception	72.22 (23.13)	81.48 (23.13)	0.5
Role function	72.92 (23.94)	66.67 (28.87)	1

*Note:* Bold values are statistical significant at *p *< 0.05.

We found a significant improvement of QoL from baseline to M3 in total score and in most of the sub‐scores, in both children and caregivers. Children reported the most significant improvement in *digestive symptoms* and *treatment burden* sub‐scores, and caregivers in *weight*, *eating disorder,* and *body image* sub‐scores, with nearly no improvement in *treatment burden*. Conversely, both reported hardly any increase in *emotional functioning* and *social/school functioning* sub‐scores.

### 3.3 Comparison of the QoL Perception as Assessed by the Patients and Their Caregivers

**Figure 1:**
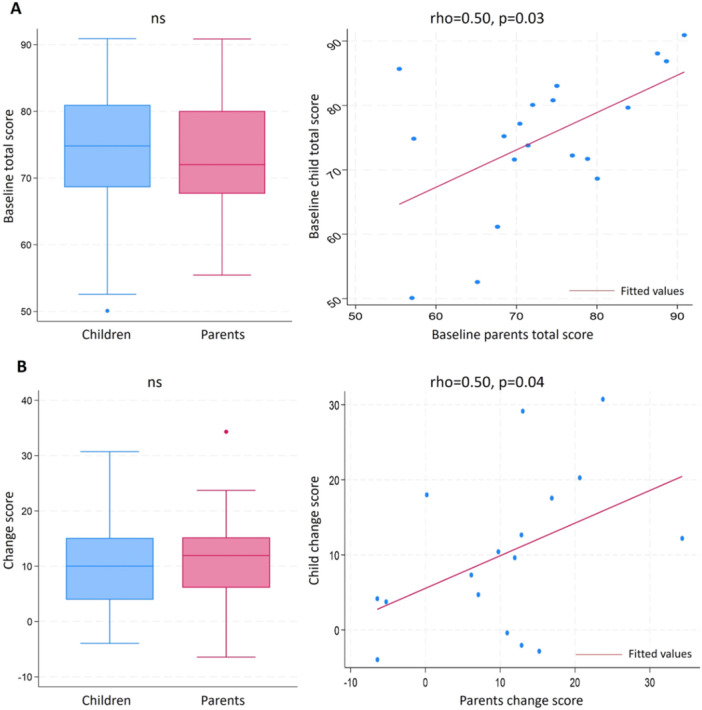
Correlation between patients and their caregivers in QoL total score at baseline (A) and change score over 3 months (B). [Color figure can be viewed at wileyonlinelibrary.com]

Despite comparable mean total scores at baseline and change scores over 3 months between children and caregivers, the correlation was moderate within a given family. The Spearman's rank correlation coefficients (rho) between the QoL perception at baseline as assessed by the patients and their caregivers varied between 0.69 and −0.06, higher for physical/symptoms domains and lower for psycho‐social domains. Significant correlations were observed for total score (rho = 0.50, *p* = 0.03) (Figure 1‐A) and for the sub‐scores *physical functioning* (rho = 0.53, *p* = 0.02), *respiratory symptoms* (rho = 0.62, *p* = 0.005), *digestive symptoms* (rho = 0.69, *p* = 0.001), *eating disorder* (rho = 0.56, *p* = 0.01), and *bogy image* (rho = 0.54, *p* = 0.02). Similarly, we observed a significant correlation between the change in total score assessed by the patients and their caregivers (rho = 0.50, *p* = 0.04) (Figure 1‐B).

### 3.4 Association Between Patients' Characteristics at Baseline and QoL Change (M3‐B)

We found a higher improvement in the total score between baseline and M3 in patients with the homozygous F508del variant compared to patients with other genotypes (*p* < 0.001). No other patient characteristic at baseline was associated with the effect of ETI on QoL (Table 3).

**Table 3:** Association between change score in patient CFQ‐R total score and covariates (demographics, clinical, radiological, functional, microbiological) using robust regression analysis.

Patient covariates	Coefficient	*p* > |t|
Sex		
Male	0.61	0.87
Female (ref)		
Age	−0.87	0.12
CFTR genotype		
F508del homozygous	12.72	< **0.0001**
Other (ref)		
CFTR modulator before the introduction of ETI		
Yes	−2.45	0.53
No (ref)		
Bronchiectasis		
Yes	−3.81	0.27
No (ref)		
Number of lobes with bronchiectasis	−1.60	0.06
Number of exacerbations in the previous year	−0.88	0.55
Expectoration		
Yes	7.21	0.10
No (ref)		
Abdominal Pain		
Yes	−3.36	0.37
No (ref)		
BMI *z*‐score	1.04	0.68
FEV_1_ *z*‐score	1.54	0.34
LCI 2.5%	−0.25	0.80
Sweat Chloride	−0.04	0.76
Pathogens identified in the airway sample		
Yes	−1.18	0.77
No (ref)		

*Note:* Bold value indicates statistical significant at *p* < 0.05.

## 4 Discussion

This is the first study focusing on QoL in children treated with ETI, given that few clinical trials reported pediatric data but restricted to the *respiratory symptoms* CFQ‐R sub‐score, and mostly in children ≥ 12 years. We report a global QoL affected at baseline and significantly improved after 3 months of ETI, as reported in studies with ETI in adolescents and adults [5, 6] and with IVA or LUM/IVA in children < 12 years old [7, 8]. Additionally, we found that only 3 months of ETI treatment improved most of the QoL domains in children, particularly concerning treatment burden from the children's perspective and digestive domains from the caregivers' perspective. Conversely, the effect of ETI on psycho‐social domains was minimal. Children and caregivers reported a similar increase in CFQ‐R total score and in most sub‐scores, despite poor agreement within a given family. Homozygote F508del genotype was associated with a greater impact of ETI on QoL in children compared to other genotypes.

Detailed results on all available specific QoL domains are described in this study, but establishing consistency with previous studies proved complicated, even in studies focusing on teenagers and adults and/or other CFTR modulators, as most of them give no insight into specific sub‐scores except *respiratory symptoms* and mostly in ≥ 12‐year‐old patients [9, 10, 11, 12, 13]. Only three studies reported detailed insights on QoL of patients treated with ETI, restricted to adult patients [14, 15] and patients ≥ 12‐year old [16], notably reporting poor improvement in digestive domains in contrast with our results in children. In the first study, with adults treated over 33 weeks with ETI, almost all domains were significantly improved, except *vitality*, *digestive symptoms*, *treatment burden,* and *social functioning* [14]. Moreover, improvement was higher in physical aspects compared to psycho‐social domains, which was also the case in our study. The second study focusing on adults showed improvement of QoL in all sub‐scores except *eating disorder*, *digestive symptoms*, *weight,* and *emotional functioning* [15]. The study focusing on ≥ 12‐year old patients showed improvements in all sub‐scores but *digestive symptoms* [16]. Several other studies in adolescents and adult patients treated with ETI showed consistent improvement in the CFQ‐R *respiratory symptoms* sub‐score, sustained over 144 weeks [13]. Additionally, a few recent studies in children aged 6−11 years old treated with ETI showed significant improvements in *respiratory symptoms* sub‐score, ranging from a between‐group difference of 5.5 points [17, 18] and 7 points [19] over 24 weeks. Studies focusing on other CFQ‐R sub‐scores were particularly scarce and didn't show unanimous results.

*Treatment burden* was the lowest sub‐score at baseline in children's questionnaires in our study, and one with the most important improvements after 3 months of ETI (Table 2). Prior to CFTR modulators era, children with CF spent approximately 75 min for treatment tasks according to Ziaian et al. [20] Several studies in adolescents and adults have shown that ETI reduces the daily treatment burden [21], allowing to de‐escalate supportive therapy without worsening in lung function in ≥ 6 year old patients [22] and ≥ 12‐year‐old patients [23].

Digestive domains of QoL (i.e., *digestive symptoms, eating disorders*, and *weight* sub‐scores) were among the most affected at baseline and the most improved after 3 months of ETI in both patients' and caregivers' questionnaires (Table 2). CF is associated with exocrine pancreatic insufficiency, diabetes, impaired enteric homeostasis, but also influences microbiome, inflammation, bicarbonate secretion, and fatty acid turnover [24]. It comes as no surprise that nutritional optimization is of high importance [25], particularly in children with potential consequences on development and active growth [26]. Numerous studies confirmed the efficacy of ETI on gastrointestinal symptoms and body weight [13, 19, 27], but few studies reported CFQ‐R sub‐scores in digestive domains. Surprisingly in adolescents and adults treated with ETI, studies showed hardly any improvement in the CFQ‐R *digestive symptoms* sub‐score, even after 12 months of treatment [14, 15, 16, 28]. In our study, children reported a weak improvement in *body image* sub‐score, which could be explained by the fact that it may be of less immediate relevance to their daily lives and need longer delay to improve [29]. Interestingly, digestive domains seemed particularly impacted by the disease, and greatly improved with ETI, in caregivers' questionnaires compared with children' questionnaires. Caregivers, struggling with their child's birth for eating cooperation, perceive eating and weight gain as an important indicator of global health and long‐term survival. Indeed, undernourished CF patients, especially the youngest, show worse clinical outcomes with reduced lung function and increased hospitalization risk and mortality [24, 25, 30, 31].

Lastly, in contrast with the clear improvement in QoL physical domains, psycho‐social domains (i.e., *emotional*, *school* and *social functioning*, and *vitality* sub‐scores) showed minimal improvement after 3 months of ETI, as previously reported in two other studies in ≥ 12‐year‐old patients [16, 32]. Recent case reports pointed out potential adverse effects of ETI on mental health, with mental fogginess, depressive and anxiety symptoms, as well as suicide attempts [33, 34]. However, two studies reported no negative impact of ETI on mental health: Piehler et al. prospectively found no modification in depressive and anxiety scores among 60 adult patients after 3 months of ETI [15]. Ramsey et al. in a study supported by Vertex Pharmaceuticals Incorporated, reported safety data from placebo‐controlled trials and postmarketing surveys along with data from the US and German CF registries and showed no worsening of depression symptoms, related‐event or prevalence [35]. This should be considered when assessing the long‐term impact of ETI on mental health. Furthermore, these QoL domains may require a longer period of treatment to achieve an improvement. Consequently, in addition to ETI treatment, patients with CF may benefit from alternative approaches to improve emotional and social QoL components. Need for such support should be assessed regularly, in patients facing numerous difficulties that come with CF and possibly with ETI, particularly in teenagers during an age full of change and questioning.

Although there seems to be a consensus in global QoL (i.e., CFQ‐R total score), it is interesting to note that patients and caregivers did not attribute equal impact of the disease and/or of ETI treatment regarding specific QoL domains (i.e., CFQ‐R sub‐scores). These differences might reflect that patients and caregivers do not internalize health changes in the same way. While parents may focus on observable behaviors or clinical milestones, younger patients may rely more on subjective experiences. Similarly, within families, there is also a notable lack of agreement in QoL scores at baseline and under ETI, notably in psycho‐social domains. Indeed, caregivers and children tend to disagree on health‐related QoL and symptoms [36], when studies suggested that agreement is higher for observable function, such as physical health, than for non‐observable function, such as emotional health [37, 38]. The differences in QoL, as evaluated by children and their caregivers, emphasize the importance of both opinions that should always be considered in order to achieve a holistic view of children's QoL.

Looking for predictive parameters of ETI effect on QoL, we showed that patients with homozygote F508del experienced higher improvement in CFQ‐R total score. We found no correlation studies between CFQ‐R total score and *CFTR* genotype. Clinical trials in ≥ 12‐year‐old patients showed no difference in CFQ‐R *respiratory symptom* sub‐score between homozygous F508del variant patients and heterozygous F508del/minimal function variants [39], nor a significant difference in non‐respiratory CFQ‐R sub‐scores [16]. One study in patients 6−11 years old treated with ETI reported no difference in CFQ‐R *respiratory symptoms* sub‐score between *CFTR* genotypes [19]. Besides QoL evaluations, clinical trials in adult or > 12‐year old patients showed a comparable effect of ETI on clinical or functional parameters and sweat test [39]. Interestingly, a strictly pediatric clinical trial reported a greater decrease in sweat chloride concentration in homozygous F508del children compared with heterozygous ones [5, 13]. Zemanick et al. hypothesized that these genotype‐related differences in children can be related to the increased abundance of the F508del‐CFTR protein that can be targeted for CFTR modulation in homozygote patients [19].

The current study has several strengths. First, it distinguished itself from prior research on QoL in CF patients treated with ETI by focusing only on children with specific QoL issues. Second, it includes a comprehensive analysis not only of global QoL or *respiratory symptoms* sub‐score but also of all available specific domains of QoL, and finally, it considers perspectives on QoL from both children and caregivers. This study also has some limitations. First, given that CF is a rare genetic disease, and that ETI was recently authorized, notably in children 6−11 years old in Switzerland, we only had access to a small sample size. Second, our patient sample was restricted to children 6−17 years with mild to severe disease, making our results difficult to generalize to the whole CF population. Conversely, the genotypic distribution of our cohort closely reflects the expected distribution among Trikafta‐eligible patients in Switzerland, according to the Swiss data of the European CF Society Patient Registry [40]. The prospective follow‐up of patients included in this study is still ongoing, integrating new patients eligible for ETI. An extended study is currently running with a larger pediatric cohort, with repeated measures of QoL at 12 months, and then yearly. This longitudinal study will enable us to confirm our results after a longer period of time, particularly with regard to disparities in QoL improvement between patients and caregivers, as well as the correlation with genotype.

## 5 Conclusions

Children treated with ETI over 3 months showed a significant improvement in their QoL, particularly in physical domains, emphasizing the importance of making ETI available worldwide for children with CF. Whilst our findings suggest that ETI substantially improves physical aspects of QoL, other domains—particularly psycho‐social components—showed limited improvement, underscoring the need for further research to understand and address these areas. Studies on QoL are of great importance, as it allows patients and caregivers to share their experience and concerns with ETI. This broadens our view of the disease, identifying domains that should be prioritized in care and research, from their point of view as expert patients.

## Author Contributions

**Sara Kümmerli:** conceptualization, methodology, data curation, investigation, writing – original draft, writing – review and editing, **Clara Fernandez Elviro:** funding acquisition, writing – review and editing, **Nicolas Regamey:** funding acquisition, writing – review and editing, **Anne Mornand:** writing – review and editing, **Mohamed Faouzi:** methodology, writing – review and editing, software, formal analysis, data curation, **Isabelle Rochat:** investigation, writing – review and editing, **Sylvain Blanchon:** conceptualization, investigation, funding acquisition, methodology, validation, writing – review and editing, project administration, supervision, resources.

## Conflicts of Interest

The authors declare no conflicts of interest.

## Data Availability

The data that support the findings of this study are openly available.
